# DNA Sequence Variants in *PPARGC1A*, a Gene Encoding a Coactivator of the ω-3 LCPUFA Sensing PPAR-RXR Transcription Complex, Are Associated with NV AMD and AMD-Associated Loci in Genes of Complement and VEGF Signaling Pathways

**DOI:** 10.1371/journal.pone.0053155

**Published:** 2013-01-15

**Authors:** John Paul SanGiovanni, Jing Chen, Przemyslaw Sapieha, Christopher M. Aderman, Andreas Stahl, Traci E. Clemons, Emily Y. Chew, Lois E. H. Smith

**Affiliations:** 1 Clinical Trials Branch, National Eye Institute, National Institutes of Health, Bethesda, Maryland, United States of America; 2 Department of Ophthalmology, Harvard Medical School, The Children’s Hospital, Boston, Massachusetts, United States of America; 3 Department of Ophthalmology, Maisonneuve-Rosemont Hospital Research Centre, University of Montreal, Montreal, Quebec, Canada; 4 Department of Ophthalmology, University Eye Hospital Freiburg, Freiburg, Germany; 5 The EMMES Corp., Rockville, Maryland, United States of America; The Ohio State Unversity, United States of America

## Abstract

**Background:**

Increased intake of ω-3 long-chain polyunsaturated fatty acids (LCPUFAs) and use of peroxisome proliferator activator receptor (PPAR)-activating drugs are associated with attenuation of pathologic retinal angiogenesis. ω-3 LCPUFAs are endogenous agonists of PPARs. We postulated that DNA sequence variation in PPAR gamma (PPARG) co-activator 1 alpha (*PPARGC1A*), a gene encoding a co-activator of the LCPUFA-sensing PPARG-retinoid X receptor (RXR) transcription complex, may influence neovascularization (NV) in age-related macular degeneration (AMD).

**Methods:**

We applied exact testing methods to examine distributions of DNA sequence variants in *PPARGC1A* for association with NV AMD and interaction of AMD-associated loci in genes of complement, lipid metabolism, and VEGF signaling systems. Our sample contained 1858 people from 3 elderly cohorts of western European ancestry. We concurrently investigated retinal gene expression profiles in 17-day-old neonatal mice on a 2% LCPUFA feeding paradigm to identify LCPUFA-regulated genes both associated with pathologic retinal angiogenesis and known to interact with PPARs or *PPARGC1A*.

**Results:**

A DNA coding variant (rs3736265) and a 3'UTR-resident regulatory variant (rs3774923) in *PPARGC1A* were independently associated with NV AMD (exact *P* = 0.003, both SNPs). SNP-SNP interactions existed for NV AMD (*P<*0.005) with rs3736265 and a AMD-associated variant in complement factor B (CFB, rs512559). PPARGC1A influences activation of the AMD-associated complement component 3 (*C3*) promoter fragment and CFB influences activation and proteolysis of C3. We observed interaction (*P*≤0.003) of rs3736265 with a variant in vascular endothelial growth factor A (*VEGFA*, rs3025033), a key molecule in retinal angiogenesis. Another *PPARGC1A* coding variant (rs8192678) showed statistical interaction with a SNP in the VEGFA receptor fms-related tyrosine kinase 1 (*FLT1*, rs10507386; *P*≤0.003). C3 expression was down-regulated 2-fold in retinas of ω-3 LCPUFA-fed mice – these animals also showed 70% reduction in retinal NV (*P*≤0.001).

**Conclusion:**

Ligands and co-activators of the ω-3 LCPUFA sensing PPAR-RXR axis may influence retinal angiogenesis in NV AMD via the complement and VEGF signaling systems. We have linked the co-activator of a lipid-sensing transcription factor (PPARG co-activator 1 alpha, PPARGC1A) to age-related macular degeneration (AMD) and AMD-associated genes.

## Introduction

Neovascular (NV) age-related macular degeneration (AMD) is a common sight-threatening disease in the elderly, accounting for more than 80% of all AMD-related vision loss in people of western European ancestry. [1] The cardinal lesions of NV AMD are proliferative growth of and exudation from vessels in the choriocapillaris, the major vascular network of the outer retina. [2], [3], [4] More than 2 million U.S. residents have advanced AMD. [5] Current treatments (intraocular injections with anti-angiogenic drugs) are a substantial financial burden on society, with direct annual medical costs reaching ∼570 million dollars. AMD-related outpatient services are incurred annually by ∼1.4 million people aged 65-and-older and contribute to ∼0.5 billion dollars in Medicare claims per year. [6] Less expensive and non-invasive treatment options for AMD are needed.

Nutrient-based approaches to AMD treatment have been focused on compounds demonstrating: 1) intake-dependent and –modifiable accretion to retinal cell types affected in AMD; and 2) biophysical and biochemical capacity to act on processes implicated in pathogenesis and pathophysiology**.** Large-scale human studies on AMD suggest a reduced likelihood of having or progressing to NV AMD among people reporting highest dietary intakes of omega-3 (ω-3) long-chain polyunsaturated fatty acids (LCPUFAs). [7], [8], [9], [10] ω-3 LCPUFAs act as key structural and signaling molecules in the retina. [11] Findings from work on *in vivo* model systems support the idea that increasing retinal tissue status of these nutrients protects against pathologic intraretinal [12], [13], [14] and choroidal [15], [16] neovascularization. The role of ω-3 LCPUFAs in cell survival and rescue is an emerging area of research, as these nutrients are precursors to families of potent neuroprotective autacoids. [17], [18], [19] Biosynthetic and cleavage enzymes, transporters, receptors, and transcriptional regulators that interact with ω-3 LCPUFAs, their precursors, metabolites, and targets are expressed in retinal areas manifesting neurodegenerative and angiogenic lesions of AMD (Institute of Human Genetics, University of Regensburg, http://www.retinacentral.org/. Accessed 2012 Nov 30). Among these are cognate lipid-sensing nuclear receptors of the peroxisome proliferator-activated receptor (PPAR) family. ω-3 LCPUFAs are endogenous PPAR agonists. [20].

PPARs act as master regulators of gene transcription and have been studied in the context of retinal vascular disease. In 2005 we first discussed the putative role of LCPUFA-PPAR relationships in the retina [11] and have recently demonstrated direct PPAR-gamma (PPARG)-mediated effects of dietary ω-3 LCPUFAs on retinal vessel formation in a oxygen-induced retinopathy (OIR) model of pathologic retinal angiogenesis. [13], [14] Use of synthetic PPAR agonists has been associated with lower likelihood and severity of pathologic retinal neovascularization. Troglitazone, a PPARG agonist, inhibited laser-induced choroidal neovascularization (a hallmark of NV AMD) in cynomoglus monkeys. [21] Fenofibrate, a PPAR activator, reduced the need for laser treatment for proliferative (neovascular) retinopathy in a large phase III clinical trial of 9795 people with type 2 diabetes. [22] The rationale for using PPAR ligands as therapeutic agents for NV AMD has been raised elsewhere. [23].

The PPARG-retinoid X receptor (PPAR-RXR) transcription complex is involved in ligand-activated transcription; this complex typically binds the *AGGTCANAGGTCA* DNA consensus sequence in peroxisome proliferator hormone response elements (PPREs) within the promoter region of target genes – when the PPAR is bound by a ligand (e.g. LCPUFAs or drugs), transcription is altered. The activity of PPARs is dependent on the shape of their ligand-binding domains and the physical interaction with co-activator and co-repressor proteins. PPAR agonists bind the PPARG-RXR transcription complex, causing a conformational shift that permits displacement of co-repressor proteins and a subsequent docking of co-activator proteins. We examined the possible influence of DNA variation in the PPARG co-activator 1 alpha gene (*PPARGC1A*), as an extension of the work on endogenous and pharmacologic PPAR agonists in pathologic retinal angiogenesis (discussed in the previous paragraph). *PPARGC1A* encodes a transcriptional regulator protein involved in constitutive activation of ω-3 LCPUFA-sensing PPARG-RXR complex target genes. PPARGC1A is a major PPARG co-activator. Our results suggest that multiple constituents (ligands and transcriptional co-activators) of the PPAR-RXR system may influence pathogenic processes implicated in NV AMD and offer promise for efficiently examining combined therapies for this blinding disease of public health significance.

## Results

**Figure 1:**
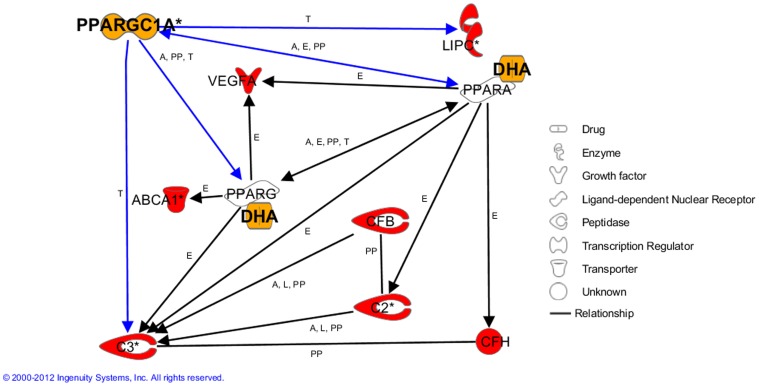
Relationships of PPARG co-activator 1 alpha (PPARGC1A) with AMD-associated genes or their products. Genes associated with AMD in extant studies are shaded in red. DHA = docosahexaenoic acid (a PPAR agonist). Diagram was generated with Ingenuity Pathway Analysis® software. Full names for genes represented by symbols exist at http://www.ncbi.nlm.nih.gov/gene/. Direct effects of PPARGC1A are represented by blue arrows. Letters on the arrows represent the nature of evidence and are defined as follows: A = activation, E = expression, L = proteolysis, PP = protein-protein interaction, T = transcription.

We examined DNA sequence variants in *PPARGC1A* for association with NV AMD in three independent U.S.-based cohorts of western European ancestry. All data are from large-scale projects designed to investigate the molecular genetics of AMD. The panel of 20 sequence variants we tested were taken from the ILLUMINA HumanCNV370v1 microarray and included 2 single nucleotide polymorphisms (SNPs) resident in exonic *PPARGC1A* regions and one resident in the 3′ untranslated region (UTR) of this gene. We applied a 4-phase approach to testing, first using our largest independent cohort as a ‘discovery’ sample, then examining the magnitude and direction of measures of NV AMD-*PPARGC1A* relationships in two other large-scale genotyping projects on AMD, and then combining measures of association with meta-analytic techniques – finally, testing these combined estimates with exact methods. After observing relationships of NV AMD with SNPs in exonic and regulatory regions of *PPARGC1A* from the meta-analysis, we examined interactions of these variants with established genetic loci for NV AMD resident in systems: 1) associated with AMD in other studies (complement, lipid metabolism, and vascular endothelial growth factor (VEGF) signaling systems); and, 2) responsive to ω-3 LCPUFA feeding in our animal models (complement cascade). **Figure 1** is a schematic of putative relationships between PPARGC1A, an ω-3 LCPUFA activator of PPARs (docosahexaenoic acid, DHA), PPARs, and genes containing AMD-associated variants (symbols for these genes are colored red).

In summary, we applied age-, sex-, and smoking-adjusted logistic regression models to analyze 843 people with NV AMD and 1032 of their elderly peers who were both AMD-free and ≥65 years-of-age. A cohort of 506 cases and 512 controls from a major university-based clinical center served as the discovery sample in phase 1. Our phase 2 replication samples consisted of 123+205 cases and 198+314 AMD-free elderly peers from 2 independent and geographically distinct university based research centers. **Table 1** contains demographic characteristics of our cohorts. After examining single locus tests for between-cohort concordance, we computed combined age-, sex-, and smoking-adjusted estimates of NV AMD-SNP relationships with meta-regression, applying random effects models to account for sample heterogeneity (phase 3). We then used max(T) permutation (10,000 iterations) on the combined samples to derive exact *P*-values for sequence variants significant at *P<*0.005 in covariate-adjusted meta-analysis (phase 4). Two-locus epistatic (SNP-SNP) interactions of AMD-associated PPARGC1A variants were then conducted with established AMD risk loci of genes in the complement, lipoprotein metabolism, and VEGF signaling systems. These genes included complement factor H (*CFH*), complement component 3 (*C3*), complement factor B (*CFB*) complement component 2 (*C2*), LIPC hepatic lipase (*LIPC*), ATP-binding cassette transporter A1 (*ABCA1*), vascular endothelial growth factor A (*VEGFA*) and its receptors fms-related tyrosine kinase 1 (*FLT1, VEGFR1*) and kinase insert domain receptor (*KDR, VEGFR2, FLK1*)).

**Table 1:** Description of Cohorts.

	Outcome	
Cohort	No AMD	NV AMD
Discovery Cohort (Michigan)		
Total, N	514	506
Mean age at exam (SE)	76.6 (0.23)	80.4 (0.30)
Female (% of cohort)	58	63
Current smoker (% of cohort)	4	7
Replication 1 (Pennsylvania)		
Total, N	198	123
Mean age at exam (SE)	76.2 (0.34)	77.5 (0.64)
Female (% of cohort)	55	58
Current smoker (% of cohort)	7	6
Replication 2 (Mayo Clinic)		
Total, N	318	205
Mean age at exam (SE)	73.7(0.34)	79.7 (0.55)
Female (% of cohort)	53	65
Current smoker (% of cohort)	6	8

Abbreviations: SE, standard error; NV AMD, neovascular AMD.

### Single Locus Tests of *PPARGC1A* Variants for NV AMD

Findings from single locus tests on *PPARGC1A* are presented in **Table 2**. A relationship emerged for rs3736265 (odds ratio (OR) = 0.63, exact *P* = 0.005), a variant in exon 9 yielding a deleterious peptide transition (SIFT score 0.01) from threonine to methionine or lysine at amino acid 612. People carrying the minor allele (AA or AG) were ∼40% less likely than their peers (GG) to have NV AMD. This variant allele was predicted with HaploReg (Broad Institute. www.broadinstitute.org/mammals/haploreg/. Accessed 2012 Nov 30) to enhance affinity to consensus sequence in hypoxia inducible factor 1 beta (aryl hydrocarbon receptor nuclear translocator, ARNT) a heterodimeric transcription factor involved with in hypoxia-induced angiogenesis. rs3736265 is in proximity to a highly conserved RNA binding domain (RNA recognition motif overlapping amino acids 678–738) involved in post-transcriptional gene expression processes (e.g. mRNA and rRNA processing, RNA stability, and RNA export). We observed relationships of the PPARGC1A intronic variant rs3755862 with NV AMD (OR = 0.61, exact *P* = 0.002); this SNP is in nearly complete linkage disequilibrium (r^2^ = 0.96) with the rs3736265 coding variant. Our inferences on a PPARGC1A-NV AMD relationship were strengthened by the observation that the 3′UTR in *PPARGC1A* also contains a NV AMD-associated variant (rs3774923, OR = 0.58, exact *P* = 0.003). As with SNP rs3736265, people carrying one or two copies of the minor allele showed a 40% reduced likelihood of having NV AMD, relative to their peers who were homozygous for the major allele. We do not have reason to believe that the rs3736265 coding SNP and the rs3774923 UTR SNP are co-inherited, based on measures of linkage disequilibrium (r^2^ = 0.64 in our analytic sample and r^2^ = 0.55 within the CEU+TSI cohorts from the International HAPMAP Project). It is important to acknowledge that the 300K SNP chip used for genotyping did not permit dense mapping of *PPARGC1A*, and thus constrained our range of inferences. Our conclusion from single-locus tests on *PPARGC1A* variants is that co-activators of PPAR-mediated processes may be reasonably implicated in pathogenesis of NV AMD.

**Table 2:** Association results of *PPARGC1A* SNPs for NV AMD in three cohorts and in meta-analysis using multivariable models.

				Discovery		Replication					
				Michigan		Pennsylvania		Mayo			
SNP	Feature	Alleles	Model	OR (95% CI)	*P*	OR (95% CI)	*P*	OR (95% CI)	*P*	OR_meta_	*P*_exact_
**rs3774923**	**3'UTR**	**A/G**	**DOM**	**0.52 (0.33–0.83)**	**0.006**	**0.69 (0.31–1.53)**	**0.181**	**0.66 (0.30–1.42)**	**0.142**	**0.580**	**0.003**
rs12650562	INTRON	T/C	ADD	1.07 (0.89–1.30)	0.459	1.16 (0.85–1.59)	0.181	1.02 (0.77–1.33)	0.453	1.074	0.380
rs7682765	INTRON	C/T	DOM	1.52 (1.02–2.28)	0.041	0.49 (0.23–1.06)	0.035	1.48 (0.84–2.60)	0.088	1.306	0.206
rs2932965	INTRON	A/G	ADD	0.97 (0.71–1.34)	0.856	0.99 (0.52–1.86)	0.486	0.99 (0.65–1.52)	0.480	0.910	0.831
rs3774921	INTRON	G/A	ADD	1.07 (0.89–1.28)	0.494	1.01 (0.74–1.38)	0.479	1.13 (0.86–1.48)	0.195	1.070	0.316
**rs3736265^a^**	**EXON**	**A/G**	**DOM**	**0.63 (0.42–0.94)**	**0.022**	**0.70 (0.34–1.46)**	**0.171**	**0.58 (0.30–1.13)**	**0.055**	**0.630**	**0.005**
rs8192678	EXON	A/G	ADD	1.02 (0.82–1.27)	0.830	1.27 (0.88–1.82)	0.101	0.88 (0.63–1.23)	0.226	1.032	0.847
**rs3755862^a^**	INTRON	**A/G**	**DOM**	**0.62 (0.42–0.92)**	**0.018**	**0.58 (0.28–1.23)**	**0.079**	**0.60 (0.32–1.14)**	**0.059**	**0.611**	**0.002**
rs2970848	INTRON	G/A	ADD	1.07 (0.86–1.32)	0.568	0.65 (0.41–1.02)	0.030	0.99 (0.71–1.37)	0.465	0.975	0.673
rs2932976	INTRON	A/G	ADD	1.26 (0.96–1.64)	0.097	0.78 (0.47–1.30)	0.170	1.07 (0.73–1.57)	0.367	1.114	0.387
rs2970853	INTRON	A/G	ADD	0.98 (0.75–1.29)	0.906	0.99 (0.62–1.56)	0.476	0.82 (0.54–1.23)	0.165	0.939	0.489
rs6448226	INTRON	G/A	DOM	0.74 (0.57–0.97)	0.029	0.91 (0.57–1.45)	0.341	1.01 (0.68–1.50)	0.486	0.832	0.127
rs7665116	INTRON	C/T	DOM	0.86 (0.64–1.16)	0.325	0.79 (0.47–1.32)	0.184	0.88 (0.56–1.38)	0.288	0.850	0.160
rs6850464	INTRON	G/A	ADD	1.01 (0.67–1.51)	0.967	0.75 (0.24–2.37)	0.310	0.69 (0.22–2.14)	0.260	0.943	0.908
rs4235308	INTRON	C/T	ADD	1.17 (0.96–1.43)	0.113	0.75 (0.51–1.10)	0.073	1.03 (0.77–1.37)	0.428	1.057	0.490
rs4550905	INTRON	G/A	ADD	0.83 (0.66–1.05)	0.115	1.07 (0.75–1.51)	0.364	1.10 (0.77–1.55)	0.303	0.939	0.391
rs4361373	INTRON	C/T	ADD	0.79 (0.52–1.20)	0.267	1.45 (0.85–2.50)	0.088	1.09 (0.61–1.94)	0.387	1.014	0.917
rs17637318	INTRON	C/T	ADD	1.06 (0.84–1.32)	0.644	1.30 (0.88–1.92)	0.093	1.02 (0.73–1.43)	0.454	1.088	0.383
rs4469064	INTRON	G/A	DOM	1.28 (0.91–1.82)	0.154	1.16(0.50–2.27)	0.329	1.27 (0.72–2.19)	0.210	1.260	0.056
rs2946385	INTRON	T/G	ADD	1.03 (0.85–1.25)	0.752	0.72 (0.50–1.04)	0.041	1.06 (0.81–1.41)	0.331	0.984	0.965

Abbreviations: 3′UTR, 3′ untranslated region; SNP, single-nucleotide polymorphism. a, SNPs in nearly complete linkage disequilibrium (r^2^ = 0.96) – no other SNPs were in linkage disequilibrium; ADD, additive model (minor allele count –2|1|0); DOM, dominant model (grouping minor allele homozygotes with heterozygotes). SNPs were tested from the panel of the ILLUMINA HumanCNV370v1 chip (SNP batch IDs at http://www.ncbi.nlm.nih.gov/SNP/snp_viewBatch.cgi?sbid=1047132). People in the reference groups (controls) were AMD-free and at least 65-years-of-age at the time of phenotype classification. We computed odds ratios (ORs) and 95% confidence intervals (95% CI) from age-, sex, and smoking-adjusted logistic regression analyses on 506 cases and 512 controls in the Discovery Cohort (University of Michigan), 123 cases and 198 controls in Replication Cohort 1 (University of Pennsylvania), and 205 cases and 314 controls in Replication Cohort 2 (Mayo Clinic, Rochester). Combined estimates (OR_meta_) were computed with age-, sex, and smoking-adjusted meta-regression – random effects models were applied in instances indicated by Cochrane’s Q statistic. All *P* values are 2-sided, with the exception of those for the replication cohorts. Exact (empirical) *P* values are from *max(T)* permutation with 10000 iterations on the full sample.

### Interaction Testing of NV AMD-associated *PPARGC1A* SNPs with AMD-associated Variants

Extant relationships depicted in Figure 1 justified our decision to examine statistical interactions of AMD-associated *PPARGC1A* variants discussed in the section above with those in genes of complement, lipid metabolism, and VEGF signaling systems. In model systems, PPARGC1A has been shown to directly activate promoter elements of *C3* [24] and *LIPC*. [25] *C3* and *LIPC* carry AMD-associated sequence variants. [26], [27] *Via* co-activation of PPARG [28], PPARGC1A may alter expression of *ABCA1* [29], [30], [31] and *VEGFA*. [32], [33] *ABCA1* and *VEGFA* carry AMD-associated sequence variants. [26], [34], [35], [36] *Via* co-activation of PPARA [37], [38], [39], [40], PPARGC1A may alter expression of CFH [41], and C2. [42] These genes carry AMD-associated sequence variants. [43], [44] In our cohorts, *PPARGC1A* SNPs showed statistical interactions with *CFB*, *C3*, *C2*, *VEGFA*, *FLT1*, and *KDR* variants. *FLT1* and *KDR* encode VEGFA receptors.

In examining interactions, we first considered single variant findings for AMD-associated SNPs in *CFH*, *C3*, *CFB*, *C2*, *LIPC*, and *ABCA1* reported in independent studies. [26], [43], [45] In our cohorts, AMD-associated loci in the complement system genes, LIPC, and ABCA1 also existed (**Table S1**). VEGF is a key molecule implicated in pathologic retinal angiogenesis variants in VEGFA have been implicated in AMD [46], [47], [48] – although, relationships have not always been replicated. [49], [50], [51], [52] While NV AMD-VEGF relationships did not exist in our cohorts for the SNPs on our microarray feature set (this may have been due to sparse coverage of the gene on the testing panel), we saw value in examining possible interactions with *PPARGC1A* SNPs.

**Table 3** contains results for NV AMD-related interactions of *PPARGC1A* SNPs significant at *P<*0.005. Most notable are findings for the *PPARGC1A* coding variant (rs3736265) with *CFB* (rs512559, *P*≤0.0046) and VEGFA (rs3025033, *P*≤0.0037). CFB influences activation and proteolysis of C3. Commentary exists in the *Discussion* on a *PPARGC1A-VEGF* interaction (*P*≤0.003) for another coding SNP in *PPARGC1A* (Gly482Ser, rs8192678) with a variant in VEGFA receptor *FLT1* (rs10507386) that changes the binding motif of the RXR consensus sequence. Inferences on the interactions of ω-3 LCPUFA-sensing PPARG-RXR complex constituents with complement and angiogenesis pathway genes in NV AMD are further strengthened by findings from: 1) large-scale population-based studies demonstrating interactions of fish (a primary source of ω-3 LCPUFAs) intake with CFH gene variants in early [53], [54] and late AMD [54]; and 2) ω-3 LCPUFA related alterations in C3 expression and attenuation of pathologic retinal neovascularization in *in vivo* systems (discussed in the section below). Our conclusion from interaction tests on *PPARGC1A* variants is that PPAR-mediated processes may be reasonably implicated to influence complement and VEGF signaling systems in the pathogenesis of NV AMD. Additional work on this concept is necessary to make conclusive inferences.

**Table 3:** Summary of interaction analysis of *PPARGC1A* SNPs and SNPs in complement and VEGF genes for NV AMD in combined cohorts.

*PPARGC1A*	Interaction SNP			
SNP (Allele)	Gene Symbol	SNP (Allele)	OR	P
**rs3736265^a^ (A)**	**CFB**	**rs512559 (C)**	**4.33**	**0.0046**
rs3755862**^a^** (A)	CFB	rs512559 (C)	4.40	0.0042
rs4235308 (C)	C3	rs2230205 (A)	0.61	0.0004
rs6448226 (G)	C2	rs638383 (A)	2.24	0.0041
rs6448226 (G)	CFB	rs512559 (C)	2.32	0.0025
rs7665116 (C)	C2	rs1042663 (A)	2.24	0.0041
rs7665116 (C)	C2	rs638383 (A)	3.21	0.0032
rs7665116 (C)	CFB	rs512559 (C)	3.37	0.0021
rs7682765 (C)	CFB	rs4151657 (C)	1.74	0.0048
rs12650562 (T)	FLT1	rs10507386 (T)	1.65	0.0049
rs2970848 (G)	FLT1	rs10507384 (G)	0.51	0.0014
**rs8192678 (A)**	**FLT1**	**rs10507386 (T)**	**1.76**	**0.0033**
rs4550905 (G)	KDR	rs2125489 (T)	1.59	0.0046
rs7682765 (C)	VEGFA	rs833069 (G)	1.80	0.0037
**rs3736265^a^ (A)**	**VEGFA**	**rs3025033 (G)**	**0.41**	**0.0037**
rs3755862**^a^** (A)	VEGFA	rs3025033 (G)	0.41	0.0035

Abbreviations: SNP, single-nucleotide polymorphism. *PPARGC1A*, PPAR gamma co-activator 1 alpha gene. Tests of SNP x SNP interactions (allelic by allelic epistasis) were conducted for *PPARGC1A* with AMD-related SNPs in complement, lipid metabolism, and, VEGF signaling genes. Text in bold type represents interactions of *PPARGC1A* SNPs in exonic regions leading to changes in protein structure. Models were based on allele dosage. Only* relationships significant at *P*≤0.005 are reported in this table. a, SNPs in nearly complete linkage disequilibrium (r^2^ = 0.96) – no other SNPs were in linkage disequilibrium. Full names for the genes listed in the ‘Gene Symbol’ column exist at: http://www.ncbi.nlm.nih.gov/gene.

### Genome-wide Expression Profiling of Murine Retinal Response to LCPUFAs

**Figure 2:**
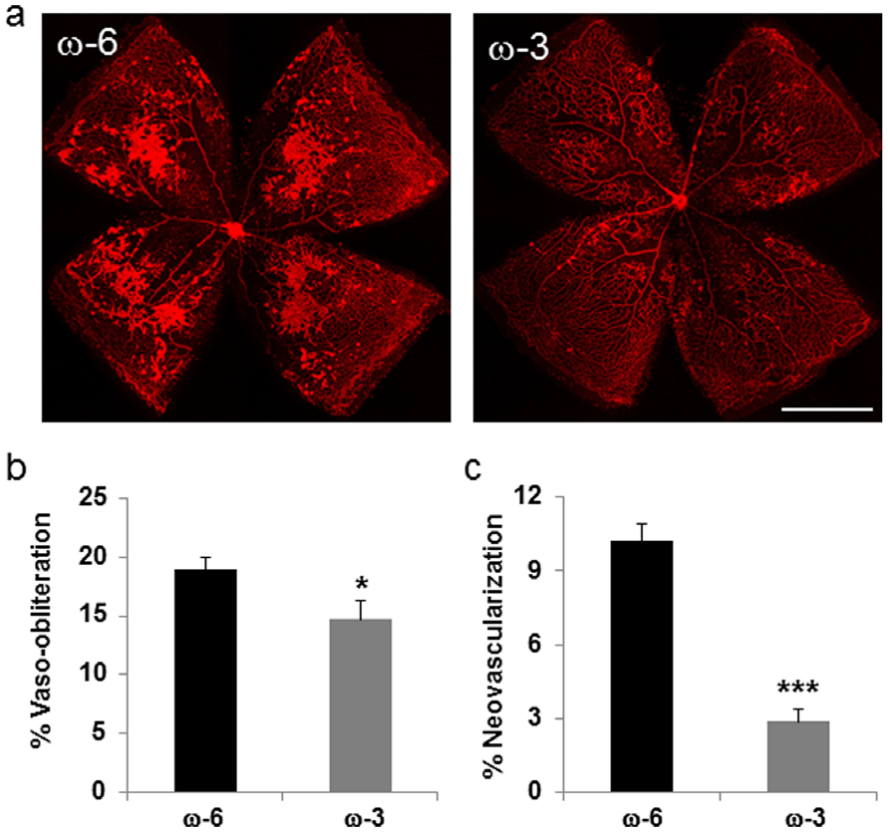
Dietary treatment of ω-3 PUFA protects against pathologic retinal neovascularization. C57 BL/6 mouse pups fed with ω-3 or ω-6 PUFA enhanced diet were exposed to oxygen-induced retinopathy. Retinas were flat mounted at postnatal day (P) 17 to visualize vasculature with contralateral retinas from the same mice isolated for gene array analysis. (**a**). Representative retina vasculature stained with isolectin B_4_ shows vaso-obliteration and pathologic neovascularization in ω-6 or ω-3 fed mice. (ω-6, n = 7 and ω-3, n = 8). Scale bar: 1mm. Quantification of (**b**) vaso-obliteration and (**c**) neovascularization in ω-6 or ω-3 fed mice. * *P*≤0.05, *** *P*≤0.001.

To consider the role of LCPUFA-regulated genes both associated with pathologic retinal angiogenesis and known to interact with PPARs or *PPARGC1A* we compared retinal gene expression profiles from an Illumina Mouse-ref 6 microarray in 17-day-old (P17) mice on a diet of 2.0% total fatty acids from ω-6 LCPUFAs (arachidonic acid, C20∶4 ω-6) to those P17 mice on a 2.0% ω-3 LCPUFA diet (1% EPA +1% DHA). Both diets were fed from time of birth. We found a 2.0-fold reduction in retinal expression of the AMD-associated *C3* gene among animals fed the 2.0% ω-3 LCPUFA diet (FDR (false discovery rate) ∼ 0.8%). Notably, identical LCPUFA exposures led to retinal tissue LCPUFA status changes and subsequent alterations in the severity of pathologic retinal angiogenesis within the OIR model. Animals receiving ω-3 LCPUFAs showed a ∼20% reduction in retinal vessel loss (*P*≤0.05) and a ∼70% reduction in pathologic retinal angiogenesis (*P*≤0.001) (**Figure 2**). [12] These findings strengthen inferences from work in humans supporting links on the influences of dietary (ω-3 LCPUFAs), pharmacologic (trolitazone, fenofibrate), and constitutive (PPARGC1A) PPAR activators as they may relate to the complement and VEGF signaling systems, and development of neovascular pathology of the retina.

## Discussion

We tested two highly conserved DNA sequence variants that code for changes in the peptide structure (rs3736265 and rs8192678) and one highly conserved SNP (rs3774923) in the regulatory 3′UTR of *PPARGC1A*; all were associated with NV AMD. An association of NV AMD with rs3736265 emerged from exact tests in age-, sex-, and smoking-adjusted models. Statistical interactions existed between rs3736265 and SNPs resident in AMD-associated loci of genes encoding key factors in complement and VEGF signaling systems. rs8192678 was associated with NV AMD through an interaction with a SNP in the VEGFA receptor FLT1. rs3774923 was related with NV AMD in single locus analyses.

Our findings extend evidence implicating endogenous (ω-3 LCPUFAs) and pharmacologic (troglitazone, fenofibrate) PPAR agonists/activators as protective agents against pathologic retinal angiogenesis to support the action of a constitutive PPAR co-activator protein (PPARGC1A) in a similar capacity. PPARGC1A may serve as a hub molecule influencing AMD pathogenesis and pathophysiology as it activates the glitazone target PPARG [28], [55], the fenofibrate target PPARA [37], [55], and C3 [24], LIPC [25], and VEGF [33] promoter fragments. Glitazones have been tested in model systems for their protective effects on retinal cell survival. [56] Troglitazone inhibited choroidal neovascularization in a *in vivo* primate model of NV AMD. [21] Fenofibrate, a synthetic PPAR activator, reduced the need for laser treatment for proliferative diabetic retinopathy. [22] Chemical structures for PPAR agonists exist at (Kanehisa Laboratories, www.genome.jp/kegg/pathway/map/map07222.html. Accessed 2012 Nov 30); those for RXR agonists and antagonists are presented at (Kanehisa Laboratories, www.genome.jp/kegg/pathway/map/map07223.html. Accessed 2012 Nov 30).

We did not observe single locus associations of NV AMD with the *VEGFA*, *FLT1*, or *KDR* variants present on our microarray test panel. However, interaction of *PPARGC1A* SNPs with those in each of these genes existed at *P*-values ≤0.005. Notable PPARGC1A-VEGF interactions emerged for coding SNPs in *PPARGC1A*: rs8192678 with a variant in *FLT1* (rs10507386, *P*≤0.003); and rs3736265 with a variant in *VEGFA* (rs3025033, *P*≤0.004). *PPARGC1A* rs8192678 is resident in the conserved consensus sequence for the EVI1 transcription factor binding site. The *FLT1* rs10507386 variant with which it shows statistical interaction also changes the binding motif of the RXR consensus sequence.

There is a link between PPARGC1A, VEGF, and the estrogen signaling system. Arany *et al*. demonstrated a PPARGC1A-dependent regulation of VEGF *via* coactivation by estrogen related receptor alpha (ESRRA) and binding to the VEGF promoter sequence. [33] Our microarray feature set did not contain ESRRA variants. We have conducted preliminary work on a network of genes encoding constituents of a signaling system with the capacity to impact VEGFA *via* PPARGC1A- and ESRRA-mediated processes. Variants in estrogen receptor 1 (ESR1, rs1999805, *P*_meta_ ≤0.002), estrogen related receptor beta (ESRRB, rs2361290, *P*_meta_ ≤0.009), and estrogen related receptor gamma (ESRRG, rs1984137 and 2820879, respective *P*_meta_ ≤0.01, 0.007) showed weak relationships with NV AMD. These genes encode proteins that interact with PPARGC1 and ESRRA and may impact VEGF signaling directly (ESR1) or via PPARs (ESRRB/ESRRG). PPARGC1A-VEGF relationships are germane to the present study since a number of drugs acting on VEGF or VEGF signaling have been tested in large-scale trials for treatment of advanced AMD [57], [58]; these include: the anti-VEGF monoclonal antibody bevacizumab [59] (Avastin), the VEGFA antibody ranibizumab [59], [60] (Lucentis), and the anti-VEGF165 aptamer pegaptanib [60] (Macugen).

We observed single locus relationships of *ABCA1* and *LIPC* SNPs on NV AMD, but no interactions with the *PPARGC1A* SNPs on our test panel. Because PPARGC1A has both the capacity to alter ABCA1 expression through its interaction with PPARG [30], [61] and to activate the LIPC promoter fragment [25], we believe this is a promising area for future work.

In conclusion, we propose that constituents of the ω-3 LCPUFA sensing PPAR-RXR axis have the capacity to act on processes impacting pathologic retinal angiogenesis *via* complement and VEGF signaling systems. A number of FDA-approved drugs targeting constituents of the axis now exist. Testing combinations of endogenous and pharmacologic PPAR agonists/activators ligands and compounds that influence PPAR co-activator proteins in pre-clinical studies on NV AMD may elucidate promising therapies for this complex blinding disease of public health significance.

## Materials and Methods

### Large-scale Genotyping Study in Elderly Humans

Data used for human genetic analyses in this report were obtained from the NEI Study of Age-Related Macular Degeneration (NEI-AMD) Database at the U.S. National Center for Biotechnology Information (NCBI) database of Genotypes and Phenotypes (dbGaP). NEI-AMD is a collaborative of researchers from the University of Michigan (Ann Arbor, MI), Mayo Clinic (Rochester, MN), University of Pennsylvania (Philadelphia, PA), and the Age-related Eye Disease Study (AREDS) group including National Eye Institute intramural investigators. Institutional review boards at each NEI-AMD study site reviewed and approved the study protocols. Each participant provided written informed consent in accordance with the *Declaration of Helsinki*.

#### Subjects and Study Design

Our analytic sample contained, respectively 506, 123, 205 people with NV AMD, and 514, 198, 318 AMD-free people (age ≥65 years) from the University of Michigan, University of Pennsylvania, and The Mayo Clinic. Details on the NEI-AMD genome-wide association (GWA) study and links to peer-reviewed publications from the project exist at: http://www.ncbi.nlm.nih.gov/gap/?term=MMAP.

#### Outcome Ascertainment

Experienced graders (ophthalmologists) classified outcomes according to AMD diagnosis in the worse eye. All participants had negative history of: 1) severe macular disease or vision loss onset prior to 40-years-of-age; 2) juvenile retinal degeneration, macular damage resulting from ocular trauma, retinal detachment, high myopia, chorioretinal infection/inflammatory disease, or choroidal dystrophy; and, 3) retinal insult that would render the fundus ungradable. Existence of neovascularization in at least one eye, according to diagnostic criteria established by the International Age-Related Maculopathy Epidemiological Study and the Modified Wisconsin Age-Related Maculopathy Grading System, was the basis for classifying people with NV AMD. In all cases of unilateral NV AMD, drusen or pigment changes also existed in the fellow eye. The likelihood of developing AMD increases 2-to-6 fold after age 75 and it was therefore essential to select our oldest AMD-free participants to minimize the potential for non-random misclassification (false negatives) in the youngest members of the control group. Our AMD-free comparison group was composed of people ≥65-years-of-age who had no large or intermediate drusen in either eye; these participants received examinations and gradings by the NEI-AMD study ophthalmologists. If small drusen or pigment changes were present in the AMD-free group, they were neither bilateral nor extensive (≤5).

#### Array-Based SNP Genotyping

All NEI-AMD specimens were genotyped with DNA microarrays at the Johns Hopkins University Center for Inherited Disease Research (CIDR, Baltimore, MD, USA) using the ILLUMINA HumanCNV370v1 chip (SNP batch IDs at http://www.ncbi.nlm.nih.gov/SNP/snp_viewBatch.cgi?sbid=1047132) with the Illumina Infinium II assay protocol. The Illumina BeadStudio Genotyping module (version 3.2.32) was used with the combined intensity of 99% of the samples to assign allele cluster definitions. The threshold for genotype calls was a gencall score ≥0.25. Reproducibility of blind duplicate samples was 99.992%. All sequence variants analyzed for the current study passed process quality and analytic filters for missingness (<5%), minor allele frequency (>1%) and Hardy-Weinberg equilibrium (HWE *P<*1 x 10^-6^ in the AMD-free group).

#### Bioinformatics

We used positional coordinates (±1000 base pairs) to analyze *PPARGC1A*, and AMD-associated variants in genes of the complement, lipid metabolism, and VEGF signaling systems. To permit a deeper inference on our findings, we used public-access databases to annotate AMD-associated variants for residence within exons, consensus sequences of highly conserved transcription factor binding sites, epigenetic marks in histone protein H3 (mono- and tri-methylation and acetylation), DNase I hypersensitivity regions, and CpG islands.

#### Statistical Analyses

We used Plink (version 1.07, http://pngu.mgh.harvard.edu/purcell/plink/) and SAS (version 9.1, Cary, NC) software for data analysis, first examining the allelic distributions of SNPs in people with NV AMD (relative to the AMD-free comparison group) with age-, sex-, and smoking-adjusted logistic regression analyses. Genotype was coded using additive, dominant (grouping minor allele homozygotes with heterozygotes), and recessive (grouping major allele homozygotes with heterozygotes) models of inheritance to obtain odds ratios (ORs) for variants within the discovery cohort (University of Michigan). Additive, dominant, and recessive models were run in the two replication cohorts. Combined ORs for single locus tests were computed across cohorts with results from each of the three models using age-, sex-, and smoking-adjusted meta-regression. Combined estimates were only computed within a given model (e.g. results from the additive model in the discovery cohort were only combined with results from the additive models run on the replication cohort). Sample heterogeneity was assessed with Cochrane’s Q statistic and random effects models were applied when indicated.

For variants attaining significance in the meta-analysis, we applied exact tests on empirical distributions of *P*-values generated with a max(T) permutation procedure set to 10000 iterations. Permutation procedures permit the computation of significance levels from empirically derived distributions. Exact *P*-values yielded by the procedure have tractable properties in obviating constraints of small sample sizes, while providing a framework for correction for multiple testing, and controlling for population substructure. In our cohorts of unrelated individuals, we swapped data values with the assumption that individuals are interchangeable under the null – this permitted construction of a new dataset sampled under the null hypothesis. Through the permutation approach only the phenotype-genotype relationship is destroyed (patterns of linkage disequilibrium between sequence variants will be preserved under the observed and permuted samples). As permutation methods sustain the correlational structure between SNPs, the approach provides a less stringent correction for multiple testing than the Bonferroni test (which assumes all tests are independent). As such, the corrected *P*-value is the relevant construct, so it is usually sufficient to apply a much smaller number of tests; resulting *P*-values ≥0.05 were considered significant.

Tests of SNP × SNP interactions (allelic by allelic epistasis) were conducted for PPARGC1A with AMD-related SNPs in complement, lipid metabolism, and, VEGF signaling genes using Plink. The analytic models were based on allele dosage. For each SNP (e.g. SNP A and B) the model took the form of Y ∼ β_0_+ β_1_A+β_2_B+β_3_AB+e. The test for interaction was based on the coefficient β_3_.

### LCPUFA Intake and Retinal Gene Expression in Mice

Our animal study adhered to the Association for Research in Vision and Ophthalmology (ARVO) Statement for the Use of Animals in Ophthalmic and Vision Research and was approved by the Children’s Hospital Boston Animal Care and Use Committee. C57BL/6J mice (stock number 000664, the Jackson Laboratory) were used for the study. Beginning at postnatal day 0 (P0), nursing mothers were fed diets enriched with either 2% ω-3 (eicosapentaenoate+docosahexaenoate) to or 2% ω-6 (arachidonate) LCPUFAs [12]. To induce vessel loss, and subsequent pathological neovascularization, nursing mothers and pups were exposed to 75% oxygen from P7 to P12 and returned to room air and sacrificed at P17. Retinas from each group were isolated and flash frozen using RNase-free techniques. Total RNA was extracted and prepared for Illumina microarray analysis using the Mouse-ref 6 chip (n = 3 biological replicates for each diet group). The chip contained ∼45,000 probe sets representing ∼34,000 genes. Microarray studies, from cDNA synthesis to raw data normalization were performed by the Molecular Genetics Core Facility at Children’s Hospital Boston. Briefly, total RNA (1 µg each) were reverse transcribed, followed by a single *in vitro* transcription amplification to incorporate biotin-labeled nucleotide, and subsequent hybridization and staining with strepatavidin-Cy3 according to the manufacturer’s instructions. Data were acquired using the Illumina BeadStudio software and analyzed for quality control, background analysis and normalization with rank invariant algorithm. Further analysis was performed using Significance Analysis of Microarray (SAM), Gene Set Enrichment Analysis (GSEA), and J-Express Pro 2.7 software. We profiled retinal gene expression with the 2% LCPUFA feeding paradigm identify LCPUFA-regulated genes both associated with pathologic NV and involved in PPAR-mediated processes.

## Supporting Information

Table S1 **NV AMD-associated variants in complement pathway genes, LIPC, and ABCA1.** Abbreviations: CHR, chromosome; SNP, single-nucleotide polymorphism. Full names for the genes listed in the ‘Gene Symbol’ column exist at: http://www.ncbi.nlm.nih.gov/gene. Combined estimates (OR_meta_) were computed with age-, sex, and smoking-adjusted meta-regression – random effects models were applied in instances indicated by Cochrane’s Q statistic. All *P* values are 2-sided.
(DOCX)
