# Supplementary material for: DNA Sequence Variants in PPARGC1A, a Gene Encoding a Coactivator of the ω-3 LCPUFA Sensing PPAR-RXR Transcription Complex, Are Associated with NV AMD and AMD-Associated Loci in Genes of Complement and VEGF Signaling Pathways
Source: PLoS One. 2013 Jan 15;8(1):e53155. doi: 10.1371/journal.pone.0053155 (PMC3546058; doi:10.1371/journal.pone.0053155)
Supplement: Table S1 — NV AMD-associated variants in complement pathway genes, LIPC, and ABCA1. Abbreviations: CHR, chromosome; SNP, single-nucleotide polymorphism. Full names for the genes listed in the ‘Gene Symbol’ column exist at: http://www.ncbi.nlm.nih.gov/gene. Combined estimates (ORmeta) were computed with age-, sex, and smoking-adjusted meta-regression – random effects models were applied in instances indicated by Cochrane’s Q statistic. All P values are 2-sided. (DOCX) [file pone.0053155.s001.docx]

Table S1. NV AMD-associated variants in complement pathway genes, LIPC, and ABCA1

| **CHR** | **Gene Symbol** | **SNP** |  | **OR_meta_** | ***P*_meta_** |
| --- | --- | --- | --- | --- | --- |
| 1 | CFH | rs800292 |  | 0.3324 | 3.23E-22 |
| 1 | CFH | rs3766404 |  | 0.3618 | 1.47E-13 |
| 1 | CFH | rs10801555 |  | 0.282 | 7.42E-40 |
| 1 | CFH | rs10754199 |  | 0.2832 | 6.16E-40 |
| 1 | CFH | rs2019724 |  | 0.3245 | 1.34E-45 |
| 1 | CFH | rs6677604 |  | 0.3343 | 3.29E-20 |
| 1 | CFH | rs419137 |  | 1.6151 | 3.84E-05 |
| 1 | CFH | rs2284664 |  | 0.3596 | 4.03E-19 |
| 1 | CFH | rs1329428 |  | 0.2751 | 9.68E-31 |
| 6 | C2/CFB | rs1042663 |  | 0.3552 | 1.19E-10 |
| 6 | C2/CFB | rs550605 |  | 0.3561 | 1.18E-10 |
| 6 | C2/CFB | rs638383 |  | 0.5128 | 0.002327 |
| 6 | C2/CFB | rs497239 |  | 0.3534 | 8.26E-11 |
| 6 | C2/CFB | rs547154 |  | 0.3536 | 9.28E-11 |
| 6 | C2/CFB | rs512559 |  | 0.5301 | 0.003528 |
| 6 | C2/CFB | rs541862 |  | 0.3534 | 8.98E-11 |
| 6 | C2/CFB | rs4151657 |  | 1.2783 | 0.0161 |
| 6 | C2/CFB | rs4151672 |  | 0.5185 | 0.000857 |
| 9 | ABCA1 | rs2066717 |  | 1.426 | 0.02423 |
| 9 | ABCA1 | rs4149281 |  | 1.4365 | 0.04984 |
| 9 | ABCA1 | rs4743764 |  | 0.8109 | 0.004201 |
| 9 | ABCA1 | rs1999429 |  | 0.627 | 0.02171 |
| 9 | ABCA1 | rs2575875 |  | 0.766 | 0.009123 |
| 15 | LIPC | rs261341 |  | 0.7448 | 0.003204 |
| 15 | LIPC | rs473224 |  | 0.7188 | 0.003421 |
| 15 | LIPC | rs261336 |  | 0.6942 | 0.000787 |
| 15 | LIPC | rs1869138 |  | 1.3458 | 0.01906 |
| 15 | LIPC | rs16940372 |  | 0.7698 | 0.02191 |
| 15 | LIPC | rs2242065 |  | 1.3907 | 0.01968 |
| 19 | C3 | rs2277984 |  | 1.3522 | 0.007949 |
| 19 | C3 | rs432001 |  | 0.6186 | 9.75E-05 |
| 19 | C3 | rs432823 |  | 0.7321 | 0.002009 |
| 19 | C3 | rs2250656 |  | 0.5561 | 7.39E-09 |
